# Comparing Genomic Signatures of Selection Between the Abbassa Strain and Eight Wild Populations of Nile Tilapia (*Oreochromis niloticus*) in Egypt

**DOI:** 10.3389/fgene.2020.567969

**Published:** 2020-10-15

**Authors:** Maria G. Nayfa, David B. Jones, John A. H. Benzie, Dean R. Jerry, Kyall R. Zenger

**Affiliations:** ^1^Centre for Sustainable Tropical Fisheries and Aquaculture, College of Science and Engineering, James Cook University, Townsville, QLD, Australia; ^2^Centre for Tropical Bioinformatics and Molecular Biology, College of Science and Engineering, James Cook University, Townsville, QLD, Australia; ^3^WorldFish, Penang, Malaysia; ^4^School of Biological, Earth and Environmental Sciences, University College Cork, Cork, Ireland; ^5^Tropical Futures Institute, James Cook University, Singapore, Singapore

**Keywords:** domestication, natural population, population genetics, population structure, outlier analysis, selection, farm management, aquaculture

## Abstract

Domestication to captive rearing conditions, along with targeted selective breeding have genetic consequences that vary from those in wild environments. Nile tilapia (*Oreochromis niloticus*) is one of the most translocated and farmed aquaculture species globally, farmed throughout Asia, North and South America, and its African native range. In Egypt, a breeding program established the Abbassa Strain of Nile tilapia (AS) in 2002 based on local broodstock sourced from the Nile River. The AS has been intensively selected for growth and has gone through genetic bottlenecks which have likely shifted levels and composition of genetic diversity within the strain. Consequently, there are questions on the possible genetic impact AS escapees may have on endemic populations of Nile tilapia. However, to date there have been no genetic studies comparing genetic changes in the domesticated AS to local wild populations. This study used 9,827 genome-wide SNPs to investigate population genetic structure and signatures of selection in the AS (generations 9–11) and eight wild Nile tilapia populations from Egypt. SNP analyses identified two major genetic clusters (captive and wild populations), with wild populations showing evidence of isolation-by-distance among the Nile Delta and upstream riverine populations. Between genetic clusters, approximately 6.9% of SNPs were identified as outliers with outliers identified on all 22 *O. niloticus* chromosomes. A lack of localized outlier clustering on the genome suggests that no genes of major effect were presently detected. The AS has retained high levels of genetic diversity (H_o_All_ = 0.21 ± 0.01; H_e_All_ = 0.23 ± 0.01) when compared to wild populations (H_o_All_ = 0.18 ± 0.01; H_e_All_ = 0.17 ± 0.01) after 11 years of domestication and selective breeding. Additionally, 565 SNPs were unique within the AS line. While these private SNPs may be due to domestication signals or founder effects, it is suspected that introgression with blue tilapia (*Oreochromis aureus*) has occurred. This study highlights the importance of understanding the effects of domestication in addition to wild population structure to inform future management and dissemination decisions. Furthermore, by conducting a baseline genetic study of wild populations prior to the dissemination of a domestic line, the effects of aquaculture on these populations can be monitored over time.

## Introduction

As aquaculture production increases, so does the number of species undergoing domestication (currently estimated at 598 species; FAO, 2018), where domestication is defined here as the adaptation of an organism from the wild to a captive environment (Price, 1984). These adaptations can be a combination of genetic changes that occur over generations through selective breeding for desirable traits (Argue et al., 2002; Hossain et al., 2011; Moss et al., 2012), but also include adjustments to a captive environment such as reduced antipredator behaviors and aggression (Johnsson et al., 1996; Robinson and Hayes, 2008).

The four main genetic processes that affect animals during domestication are founder effects, selection, genetic drift, and inbreeding (Ladizinsky, 1985; Clutton−Brock, 1992; Ollivier, 2002; Mignon-Grasteau et al., 2005; Andueza-Noh et al., 2015); however, the extent of their effects on the genome can vary. In general, the consequences of inbreeding and genetic drift are widespread and can be observed throughout the genome, whereas selection tends to act differentially across the genome depending on the genetic architecture of the trait (Burke et al., 2005). These micro-evolutionary processes need to be taken into consideration when trying to identify how an organism’s genome is being affected by domestication.

One way to understand the genetic consequences of domestication and to identify signatures of selection is to compare population genetic metrics between captive and wild populations (Simmons et al., 2006; López et al., 2019). Recent advances in high-throughput whole genome sequencing has enabled the cost-effective development of genome-wide markers for many non-model species. Such technological developments have enabled researchers to not only harness increased power in identifying the extent to which genetic processes like selection, genetic drift, and inbreeding affect a genome, but also identify specific regions of the genome that have responded to such processes (Carter et al., 2008; Scandura et al., 2011; López et al., 2019). Therefore, evaluating the genetic differences between wild and domestic populations can also help identify genomic regions associated with domestication and desirable market traits, wild populations that exhibit these traits, and local adaptations in wild populations. Additionally, these differences can be used to detect escapees and help estimate their potential impact on local populations.

In 2002, the Abbassa Strain (AS) of Nile tilapia (*Oreochromis niloticus*) was initiated by the WorldFish Center in an effort to increase aquaculture production of this species in Egypt (Rezk et al., 2009; Ibrahim et al., 2013). Its purpose was to provide a genetically diverse population based on the local strain of Nile tilapia that could be selectively improved for growth. Subsequently, the AS was established from four Egyptian populations (three wild: Zawia, Abbassa, and Aswan; one hatchery: Maryout). The production of AS is currently restricted to the Nile Delta; however, WorldFish and the Egyptian government plan to disseminate the AS line throughout Egypt.

To date, genetic diversity studies have found that wild Nile tilapia populations have evidence for sub-structuring in Egypt, particularly between populations in the Nile Delta in Upper Egypt compared with populations in the Lower Egyptian portion of the Nile River (Hassanien et al., 2004; Hassanien and Gilbey, 2005). However, due to the age of the studies, possible translocations and the availability of improved genetic technologies, updated investigations into the genetic structure of these populations using high density, genome-wide markers are warranted to determine the current status of wild population genetic structuring.

This study investigated the population genetic structure, evidence for signatures of selection, and genetic diversity related to domestication in the AS compared to wild Egyptian Nile River *O. niloticus* populations. This information can then be used to understand the impact disseminating the AS may have on wild stocks, as well as understand if targeted breeding in the AS has resulted in signatures that may be indicative of domestication.

## Materials and Methods

### Sampling and DNA Extraction

#### Wild Population Sampling

Fin clips from 400 Nile tilapia were collected from eight wild populations (Aswan, *n* = 50; Manzala Lagoon, *n* = 50; Kanata, *n* = 50; Lake Idku, *n* = 50; Damietta, *n* = 50; Lake Burullus, *n* = 50; Rosetta, *n* = 50; and Asyut, *n* = 50) along the Nile River, Egypt. Of these, Aswan was one of the four sites from which individuals were sampled to create the domesticated Abbassa Strain in 2002. Samples were obtained directly from commercial fishing boats, with fish for an individual location obtained over a distance of approximately 1 to 175 km. Samples were preserved in 70% ethanol and submitted to Diversity Arrays Technology (DArT) in Canberra, Australia, for DNA extraction and high throughput genotyping by sequencing using proprietary DArTseq^TM^ technology^1^. To obtain purified DNA, extractions were conducted using commercially available extraction kits (Promega, Qiagen; Lind et al., 2017).

#### Abbassa Strain Population Sampling

Fin clips from 483 samples were collected from the three most recent generations of the AS at the time of this study [121 individuals from generation 9 (G9); 216 individuals from generation 10 (G10); and 146 individuals from generation 11 (G11)]. DNA extractions and genotyping were conducted by Diversity Arrays Technology (DArT) as described in Lind et al. (2017).

### Library Preparation and Sequencing

DArTseq^TM^ uses a combination of complexity reduction methods, which were originally optimized on the Jaccoud et al. (2001) microarray platform. These methods effectively select low copy sequences from a genome before sequencing them on next generation sequencing platforms (Kilian et al., 2012; Courtois et al., 2013; Von Mark et al., 2013; Raman et al., 2014; Lind et al., 2017). As this process uses both a rare and a more frequently cutting enzyme, it is similar to double digest RAD sequencing (ddRAD; Peterson et al., 2012; Lind et al., 2017).

DArTseq^TM^ reduced-representation libraries were prepared as described by Sansaloni et al. (2011) and Kilian et al. (2012). In short, optimization of the complexity reduction process for Nile tilapia was achieved by using a combination of PstI and HpaII methylation-sensitive restriction enzymes for digestion and unique barcode sequences ligated onto the ends of each resulting fragment (Kilian et al., 2012; Lind et al., 2017; Kjeldsen et al., 2019). Bridge amplification was achieved by incorporating a PstI specific adaptor with an Illumina flow-cell attachment region, primer sequence, and unique barcode coupled with the reverse HpaII specific adaptor containing a second Illumina flow-cell attachment sequence (Lind et al., 2017; Schultz et al., 2018; Kjeldsen et al., 2019). Therefore, only fragments containing both PstI and HpaII cut sites were amplified for sequencing. To ensure complete digestion and a uniform range of fragment sizes, all samples were checked using an agarose gel. Any samples which displayed downshifted bands after digestion during DArTseq library preparation were removed. These downshifted samples exhibited a lower amplicon range than expected when compared to other samples and are not ideal for a consistent genotype assay. A total of eight downshifted samples were not included within the sequencing effort. Additionally, a minimum of 15% random technical replicates were included in all genotyping batches for quality control.

### Quality Control and Initial SNP Calling

DArT’s proprietary marker calling algorithm DArTsoft14 was used to call SNPs (Lind et al., 2017), implemented in the KDCompute framework^2^. Samples from wild locations were then co-analyzed by DArT alongside 483 samples from three generations of the AS, which had already been processed using DArTseq^TM^ technology as part of a previous experiment (Nayfa et al., 2020).

A total of 19,505 SNP markers were identified across all 875 samples and were filtered using a custom Python script adapted from DartQC^3^ and CD-HIT-EST (Li and Godzik, 2006). Briefly, samples with greater than 50% missing data were removed from the dataset and individual genotypes calls made with fewer than five reads were silenced. Genotypes with a count comparison, or the comparison of read counts between REF and SNP alleles, were silenced if they fell between 0.05 and 0.1, where <0.05 is considered to be homozygous and >0.1 is considered to be heterozygous (see text footnote 3). SNPs were then filtered if they had an average replication statistic of less than 90%, a call rate less than 50%, and a minor allele frequency (MAF) of less than 1% in at least one population. The clone ID sequences from which SNPs were called and clustered together at 95% similarity using CD-HIT-EST (Li and Godzik, 2006). Within each cluster, the SNP with the highest MAF was retained to ensure a more even representation of the genome. A total of 9,827 high quality SNPs and 821 samples (90.9% of collected samples) were retained for all downstream analyses.

### Population Genetic Structure

#### Broad Scale Population Structure

To determine broad-scale population differentiation across the eight wild locations and three generations of the AS, two separate clustering models (the allele frequencies correlated model and the allele frequencies independent model) were utilized within a Bayesian cluster population structure analysis in STRUCTURE 2.3.4 (Pritchard et al., 2000; Falush et al., 2003, 2007; Hubisz et al., 2009). In order to avoid inappropriate clustering due to *K* being set too small, *K* was set from 1 to 12, so that the maximum clustering possible was larger than the number of putative populations (Kalinowski, 2011). Three repeat runs were performed for each *K* (1–12), with a burn-in period of 5,000 iterations followed by 50,000 final iterations using the admixture model and no prior probabilities for cluster membership. Both clustering models yielded near identical results. The optimal number of population clusters, *K*, was determined using an ad hoc statistic *Delta K* (Δ*K*). Δ*K* is the degree of change in the log probability of data between successive *K* values, and was calculated using Structure Harvester (Evanno et al., 2005; Earl and vonHoldt, 2012). To ensure that any structuring observed in the wild populations was not biased by the inclusion of individuals from a domesticated line, analyses with the same parameters were repeated on only the eight native sampling locations testing a *K* of 1 to 9.

#### Fine Scale Population Structure

Fine-scale population genetic structuring across all eight wild sampling locations and the three AS generations was assessed using pairwise relationships based on identity-by-state (IBS) distance calculated in Plink v.1.9 (Purcell et al., 2007; Purcell, 2020). These relationships were then visualized using mutual k-nearest neighbor graphs in the NETVIEW pipeline v.1.1 at kNN values between 1 and 100 (Neuditschko et al., 2012; Steinig et al., 2016). To confirm fine-scale population genetic structuring a principal coordinates analysis (PCoA) was also conducted using GenAlEx v. 6.51b2 (Peakall and Smouse, 2006, 2012). Genetic distance per population was first calculated in GenAlEx v. 6.51b2 and then a covariance-standardized PCoA method was applied (Peakall and Smouse, 2006, 2012). To identify the percentage of genetic variation that can be attributed to differences between and within populations, an analysis of molecular variance (AMOVA) based on genetic distance was then conducted using 9,999 permutations in GenAlEx v. 6.51b2 (Peakall and Smouse, 2006, 2012).

To test if any identified genetic structuring followed an isolation-by-distance model of population divergence, Mantel’s test for correlation between genetic (*F*_st_) and genetic distance (km) was conducted in the R package *adegenet* using 10,000 permutations in the *mantel.randtest()* function (Jombart, 2008; Jombart and Ahmed, 2011). Genetic distance was calculated using an Euclidean method based on Angular distance in the *adegenet* function *dist.genpop()* (Jombart, 2008; Jombart and Ahmed, 2011). Geographic distances were calculated based on the shortest distance between two points according to the “Vicenty (ellipsoid)” method calculated using the R package *geosphere* (Hijmans et al., 2017).

### Signatures of Selection

#### Population Outlier Analysis

To identify outliers (including loci which are being influenced by selective processes), two independent software were utilized: Arlequin 3.5.2.2 (Excoffier and Lischer, 2010) and BayeScan 2.1 (Foll and Gaggiotti, 2008; Foll, 2012). For comparisons between the two groups (wild Nile tilapia and the domesticated AS Nile tilapia), only those candidate outliers that were jointly identified between programs were categorized as putative outliers. Outlier analyses within Arlequin 3.5.2.2 were based on a hierarchical island model with 20,000 simulations, 50 simulated groups, and 100 demes simulated per group (Excoffier and Lischer, 2010). AMOVA computations were conducted using a pairwise difference method with no Gamma correction (Excoffier and Lischer, 2010).

Outlier analyses within BayeScan 2.1 were based on a neutral model with 1:10 prior odds, 20 pilot runs consisting of 5,000 iterations each, followed by 100,000 iterations with a burn-in-length of 50,000 iterations as recommended by Foll (2012). To establish whether a neutral or selection model was in effect for each SNP the ratio of posterior probabilities, Bayes factors (BF) were calculated. A Jeffrey’s interpretation of “strong” BF (*p-value* ≤0.05) to “decisive” BF (*p-value*- ≤0.01) was then utilized to identify outliers and ascertain which model the posterior odds favored (Foll, 2012). For markers which fell under a selection model, positive alpha values were then used to identify markers that were under diversifying or directional selection, whereas negative alpha values were used to identify those markers under background, or balancing, selection (Foll, 2012). For pairwise comparisons of populations, only BayeScan as (1) the hierarchical method utilized in Arlequin required the use of multiple populations per grouping, this analysis did not support individual pairwise population comparisons and (2) the majority of outliers in genetic clusters identified by BayeScan were also identified by Arlequin 3.5.2.2 (approximately 70%).

To test for the normality of markers, quantile-quantile plots (QQ-plots) with a 95% confidence interval were constructed in the R package GWASTools v. 3.1 (Gogarten et al., 2012) for the full marker set, as well as the neutral marker sets (Gondro et al., 2013; Hayes, 2013). To validate the outlier selection criteria selected (i.e., markers jointly identified by both BayeScan and Arlequin), QQ-plots using the two different neutral marker sets (one with all identified outliers removed and one with only jointly identified outliers removed) were created. How well the data fitted the assumption of normality was then compared between both datasets, and only those jointly identified moved forward. Comparison of these datasets allowed the validity of identified outliers to be established.

#### Genomic Regions Under Selection

Raw clone sequences from which SNPs were identified during the DArTseq process were annotated to the available genome assembly for *Oreochromis niloticus* (GenBank Assembly Accession: GCA_00188235.2; Orenil1.1) using a custom Perl script based on NCBI CGI BLAST interface with a 70% minimum sequence identity (Heller-Uszynska et al., 2011; Supplementary Material 1). The Orenil1.1 genome assembly was used instead of the more recent O_niloticus_UMD_NMBU assembly as it was in greater agreement with the linkage maps created for the Abbassa Strain (unpublished data).

### Genetic Diversity Statistics

To determine the genetic diversity available within each sampled population (wild and AS), observed (*H*_*o*_) and expected (*H*_*e*_) heterozygosity in addition to the number of polymorphic markers within a population were calculated in ARLEQUIN 3.5.2.2 (Excoffier and Lischer, 2010). Heterozygosity and the number of polymorphic markers were examined across scenarios with different amounts of missing data (all markers; 5% missing allowed per SNP within individual populations; and 50%, 25%, and 5% missing allowed per SNP across the entire dataset). Additionally, average multilocus heterozygosity (MLH) for each population was computed using the R package inbreedR (Stoffel et al., 2016). Private SNPs per population were calculated using the R package PopGenKit v.1.0 (Paquette and Paquette, 2011). To determine the level of differentiation amongst populations, pairwise and global *F*_*st*_ values were calculated in ARLEQUIN 3.5.2.2 (Excoffier and Lischer, 2010). Levels of inbreeding per sampling location and time point were examined using the inbreeding coefficient (*F*_is_) calculated in ARLEQUIN 3.5 using 1,000 permutations (Excoffier and Lischer, 2010). Hardy-Weinberg equilibrium (HWE) was calculated in ARLEQIUN 3.5 using 1,000,000 Markov chain steps and 100,000 dememorization steps (Excoffier and Lischer, 2010; Waples, 2014). Effective population size in each native location was calculated using the linkage disequilibrium method (LDN_e_) in NeEstimator V2.01 (Do et al., 2014).

## Results

### Population Genetic Structure

#### Broad Scale Population Structure

**FIGURE 1:**
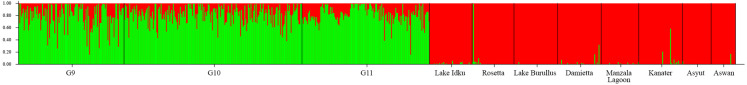
Broad-scale population structure. Structure plot of the three AS generations and the eight wild sampling locations at *K* = 2. Colors (green and red) represent the two genetic clusters identified. Vertical bar colors are indicative of admixture of the two genetic clusters per individual. The wild sampling locations are ordered via geographical distance order.

The ad hoc Δ*K* statistic indicated evidence for two major genetic clusters within the dataset (Supplementary Material 2). This distinction was supported by STRUCTURE admixture analysis whereby the domesticated AS generations formed one genetic cluster and the eight wild populations comprised the second cluster (Figure 1 and Supplementary Material 2). With a *K* of two, the admixture model used in STRUCTURE assumes that each individual has ancestry from only one or both of these genetically distinct clusters (Lawson et al., 2018). Given this, every individual from the AS shares genetic material with the wild Nile tilapia. This is reflected in the minimal population structuring identified between AS and wild sampling locations identified by pairwise *F*_*st*_ values (*F*_*st*_ = −0.008–0.058; Supplementary Material 3). The largest genetic distance was observed between the two most southern wild sampling locations (Asyut and Aswan) and the AS (*F*_*st*_ = 0.045–0.058; Supplementary Material 3).

Within the wild sampling locations, one individual from Rosetta was more closely related to the AS than to the wild genetic cluster (Figure 1). There are two individuals from Damietta, two from Kanater, and one from Aswan which also had a higher proportion of shared ancestry with the AS than expected based on the other individuals in the wild genetic cluster (Figure 1).

**FIGURE 2:**
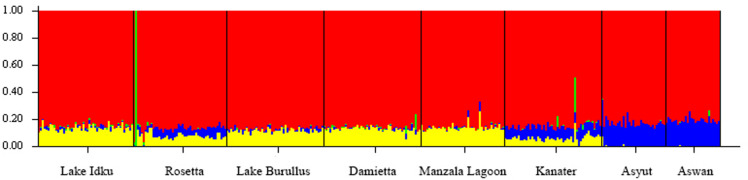
Broad-Scale Population Structure. Structure plot of the eight wild sampling locations along a geographical gradient down the Nile River, Egypt at *K* = 4. Colors (red, yellow, blue, and green) represent the four genetic clusters identified. Vertical bar colors are indicative of admixture of the four identified genetic clusters per individual.

When the eight wild locations were examined separately, the Δ*K* statistic identified a total of four weakly separated genetic clusters (Figure 2 and Supplementary Material 4). While each sampled location showed evidence of all four genetic clusters within them, the proportion of these genetic clusters changed along the northern to southern gradient of the Nile River. The two most southern populations (Asyut and Aswan) exhibited the greatest difference in admixture ratios compared to Lake Idku, Rosetta, Lake Burullus, Damietta, and Manzala Lagoon (Figure 2). Kanater displayed the largest shift between the northern and southern sampling locations (Figure 2). This was supported by pairwise *F*_*st*_ values which revealed no subpopulation structuring amongst the wild populations. The greatest *F*_*st*_ was between the northernmost population (Lake Burullus) and the southernmost population (Aswan; *F*_*st*_ = 0.021; Supplementary Material 3).

Individuals showing an independent genetic cluster (green) in Figure 2 were the same as individuals which displayed a greater association with the AS in Figure 1. This pattern suggests these individuals are possibly escapees (Rosetta) or subsequent offspring (Kanater, Damietta, and Aswan) of the AS.

#### Fine-Scale Population Structuring

**FIGURE 3:**
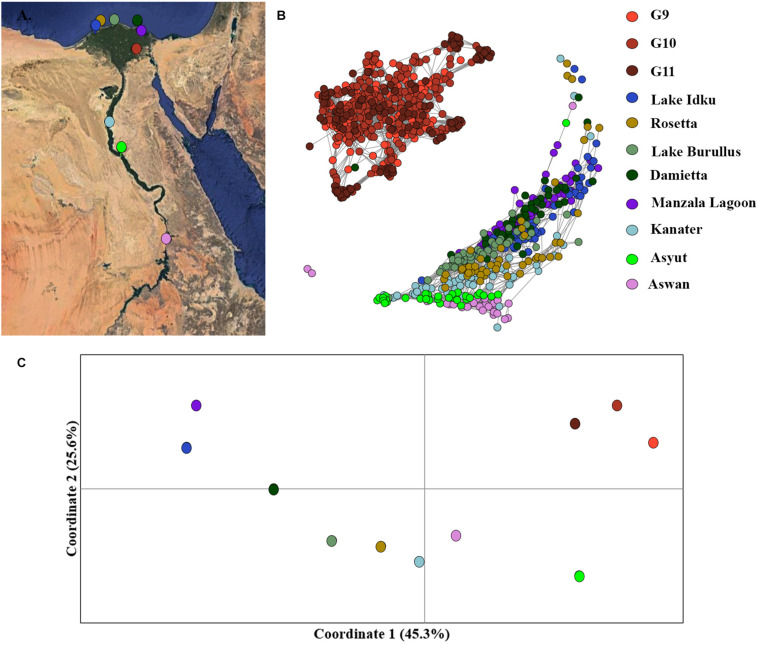
Fine-scale population structuring. **(A)** Map of sampling locations along the Nile River in Egypt. **(B)** Population clustering of all populations using an identity-by-state matrix constructed using the NETVIEW v1.1 pipeline at kNN = 20. **(C)** Population clustering based on population genetic distance using a principal coordinates analysis (PCoA). Coordinates 1 (45.3%) and 2 (25.6%) account for a total of 70.9% of the variation among populations, with coordinate 3 (not displayed) accounts for 11.3 % of the variation.

Mutual k-nearest neighbor analyses conducted in NetView pipeline v.1.1 to determine fine-scale population structuring exhibited a similar pattern to the STRUCTURE admixture analysis. The three generations of the AS formed a distinct genetic cluster separate from the eight wild sampling locations, whilst the wild populations exhibited evidence of isolation-by-distance (Figure 3B and Supplementary Material 5). The two most southern populations (Asyut and Aswan) were distinguishable from the populations further north and form a smaller, separate cluster (Supplementary Material 5). However, a few individuals from these southern locations intermingled with northern samples indicating gene flow between these populations (Figure 3B). There is a single sample from Damietta which clustered with the AS. Since Damietta is in close geographical proximity to the farm, it is conceivable that this individual is an escapee from the AS program (Figure 3B). Additionally, two individuals from the southernmost Aswan population formed a third clustering: indicating, that populations from further south in the Nile River and connecting waterways and lakes may exhibit greater variation amongst populations or include hybridized individuals (Figure 3B).

Similar patterns were observed in the PCoA analysis of population, the three AS generations formed an independent cluster along the first coordinate axis which accounted for 45.3% of the variation among clusters (Figure 3C). An AMOVA between the three generations of AS and the eight wild populations indicated that 13.8% of the molecular variance observed was between the AS and wild genetic clusters (*p* = 0.0001). The Nile Delta populations were more distinct from the upstream Nile populations, Asyut and Awan (Figure 3C). Kanater is located both physically and genetically between the five Nile Delta populations and the two southernmost upstream populations (Figures 3A,B). An AMOVA of the eight wild populations demonstrated that 10.6% of the molecular variance observed is among populations (*p* = 0.0001).

### Signatures of Selection

The QQ-plots examining the entire marker set revealed that the data violated the assumption of normality, indicating the presence of outliers (Supplementary Material 6). A total of 674 outliers were jointly identified by both BayeScan and Arlequin between wild and domestic genetic clusters (Table 1). These outliers were confirmed by re-examining normality of the data using QQ-plots when the identified outliers were removed. QQ-plots revealed that the data conformed more to the assumption of normality than previously; however, there were likely still unidentified outliers in the dataset (Supplementary Material 6). When all outliers identified by either BayeScan or Arlequin were removed from the dataset, they did not conform to the assumption of normality, indicating that those outliers identified by only one program were unlikely to be true outliers (Supplementary Material 6). This confirmed the decision to utilize only jointly identified markers by both BayeScan and Arlequin when multiple sampling sites constituted a population (i.e., domestic or wild genetic clusters).

**TABLE 1:** Pairwise outlier analysis all directional and balancing outlier loci identified.

	Gen 10	Gen 11	Lake Idku	Rosetta	Lake Burullus	Damietta	Manzala Lagoon	Kanater	Asyut	Aswan	Wild
Gen 9	0	11	5	3	11	4	6	6	13	6	
Gen 10		14	6	6	13	3	5	4	11	5	
Gen 11			2	4	13	5	5	4	10	1	
Lake Idku				0	0	0	0	0	1	0	
Rosetta					2	0	0	0	11	2	
Lake Burullus						0	3	1	0	1	
Damietta							1	0	4	0	
Manzala Lagoon								0	3	0	
Kanater									1	1	
Asyut										3	
Domestic											674

*Outliers detected between the identified broadscale populations (i.e., Wild and Domestic) are those that were jointly identified by both BayeScan and Arlequin. Outliers detected between pairwise comparisons of sampled locations and AS generations are those detected by BayeScan, the more conservative of the two programs utilized for analysis.*

The greatest number of outliers (674) was found between the two genetic clusters identified using broad-scale population structuring analysis (Table 1 and Figure 1). Of those outliers, 187 had negative alpha values in BayeScan and are under balancing selective forces, whereas the remaining 487 outliers had positive alpha values indicating directional selection. On average, pairwise comparisons of either Asyut or Lake Burullus to domestic populations (G9-11) yielded the greatest number of outliers (10–13; Table 1). The five wild populations which are most closely located in the Nile Delta (Rosetta, Lake Burullus, Damietta, Manzala Lagoon, and Kanater) had the fewest identified outliers (zero-three) when compared pairwise amongst themselves (Table 1). Regarding the pairwise comparisons of wild populations, Asyut vs. Rosetta had the greatest number of outliers (11) followed by Asyut vs. Damietta (4; Table 1).

Outliers accounted for approximately 6.9% of the entire SNP marker set, with balancing outliers accounting for approximately 1.9% of the entire marker set and diversifying outliers accounting for approximately 5.0%. Diversifying outliers accounted for 72.3% of all identified outliers, whereas balancing outliers accounted for 27.7% (Supplementary Material 7). Of the 674 identified outliers, 493 mapped back to the Orenil1.1 genome (Supplementary Material 7). Every chromosome in *O. niloticus* had both directional and balancing outliers present, with the number of outliers per chromosome ranging from 9 to 61 (Supplementary Material 7).

### Genetic Diversity

The estimated effective population size for the AS ranged between N_e_ = 14.8–48.6 per generation, with only approximately 20 - 81% of each generation’s breeding population genotyped (**Table 4**.2; Nayfa et al., 2020). Estimated effective population sizes of wild populations ranged from 30.5 – infinite, with infinite being indicative of an infinite-sized ideal population and is taken to be an extremely high and positive value (**Table 4**.2; Jones et al., 2016). Despite these variations in effective population size, all *F*_is_ values were non-significant and negative in all AS generations and wild populations (Supplementary Material 8). The proportion of SNPs that deviated from HWE in domestic populations were 2.8 – 14.6 times more frequent than in wild populations (Supplementary Material 8).

Overall, the domestic population genetic cluster had higher expected heterozygosity (H_e_), observed heterozygosity (H_o_), multilocus heterozygosity (MLH), minor allele frequencies (MAF) and polymorphic loci than the wild genetic cluster when all, neutral, or directional markers were taken into consideration (Table 2). The greatest difference among populations and genetic clusters was observed when directional outlier markers were examined. When AS generations and wild population were individually considered, levels of both H_o_ and H_e_ for all and neutral markers were similar. In most instances, wild sampling locations (except H_o_: Rosetta and Damietta and H_e_: Rosetta, Damietta, and Kanater) had higher levels of heterozygosity than individual AS generations (Table 2). Rosetta had the lowest observed heterozygosity (H_o_All_ = 0.181, H_o_Neutral_ = 0.180, and H_o_Directional_ = 0.154) and expected heterozygosity (H_e_All_ = 0.212, H_e_Neutral_ = 0.210, and H_e_Directional_ = 0.214) in these three marker sets (Table 2).

**TABLE 2:** Genetic diversity indices calculated using all SNPs and subsets of SNPs (neutral markers, directional outlier markers, and balancing outlier markers).

	Category	*n*	N_eLD_	Ho ± SE				He ± SE				MLH ± SE				MAF ± SE				Polymorphic Loci			
								
				All	Neutral	Direc- tional	Balan- cing	All	Neutral	Direc- tional	Balan- cing	All	Neutral	Direc- tional	Balan- cing	All	Neutral	Direc- tional	Balan- cing	All	Neutral	Direc- tional	Balan- cing
Gen 9	Domestic	121	34.1–45.6	0.211 ± 0.013	0.207 ± 0.013	0.271 ± 0.013	0.284 ± 0.014	0.232 ± 0.014	0.226 ± 0.014	0.329 ± 0.012	0.291 ± 0.014	0.182 ± 0.002	0.178 ± 0.002	0.221 ± 0.005	0.271 ± 0.003	0.191 ± 0.006	0.126 ± 0.031	0.249 ± 0.014	0.203 ± 0.011	9,291	8,658	447	186
Gen10	Domestic	204	37.9–48.6	0.212 ± 0.010	0.206 ± 0.010	0.283 ± 0.010	0.280 ± 0.011	0.231 ± 0.011	0.224 ± 0.011	0.338 ± 0.009	0.290 ± 0.011	0.170 ± 0.002	0.166 ± 0.002	0.214 ± 0.003	0.255 ± 0.003	0.178 ± 0.004	0.127 ± 0.028	0.242 ± 0.014	0.206 ± 0.013	8,671	8,084	406	181
Gen 11	Domestic	145	14.8–21.9	0.211 ± 0.013	0.205 ± 0.012	0.283 ± 0.012	0.282 ± 0.013	0.229 ± 0.013	0.222 ± 0.013	0.339 ± 0.011	0.289 ± 0.012	0.175 ± 0.001	0.171 ± 0.001	0.222 ± 0.004	0.268 ± 0.003	0.185 ± 0.005	0.126 ± 0.022	0.247 ± 0.011	0.204 ± 0.010	8,934	8,338	414	185
Lake Idku	Wild	49	493.9-Infinite	0.214 ± 0.024	0.213 ± 0.023	0.200 ± 0.023	0.286 ± 0.024	0.232 ± 0.024	0.229 ± 0.024	0.247 ± 0.025	0.284 ± 0.022	0.133 ± 0.002	0.132 ± 0.002	0.102 ± 0.002	0.270 ± 0.005	0.156 ± 0.007	0.129 ± 0.022	0.153 ± 0.030	0.200 ± 0.020	6,404	5,970	251	183
Rosetta	Wild	48	30.5-Infinite	0.181 ± 0.029	0.180 ± 0.029	0.154 ± 0.029	0.274 ± 0.028	0.212 ± 0.029	0.210 ± 0.029	0.214 ± 0.031	0.286 ± 0.027	0.134 ± 0.002	0.133 ± 0.002	0.109 ± 0.002	0.261 ± 0.007	0.168 ± 0.009	0.125 ± 0.022	0.157 ± 0.028	0.199 ± 0.020	7,626	7,076	366	184
Lake Burullus	Wild	50	746.6-Infinite	0.221 ± 0.024	0.220 ± 0.024	0.208 ± 0.024	0.286 ± 0.023	0.236 ± 0.024	0.233 ± 0.024	0.251 ± 0.025	0.287 ± 0.022	0.142 ± 0.001	0.141 ± 0.001	0.110 ± 0.003	0.276 ± 0.004	0.169 ± 0.007	0.127 ± 0.022	0.144 ± 0.029	0.200 ± 0.020	6,754	6,285	285	184
Damietta	Wild	50	590-Infinite	0.202 ± 0.023	0.201 ± 0.023	0.180 ± 0.023	0.272 ± 0.023	0.225 ± 0.024	0.223 ± 0.024	0.226 ± 0.025	0.285 ± 0.022	0.136 ± 0.002	0.135 ± 0.002	0.107 ± 0.003	0.257 ± 0.005	0.166 ± 0.007	0.127 ± 0.024	0.149 ± 0.029	0.203 ± 0.021	7,041	6,551	307	183
Manzala Lagoon	Wild	43	175.4-Infinite	0.222 ± 0.025	0.221 ± 0.025	0.205 ± 0.025	0.271 ± 0.025	0.240 ± 0.025	0.238 ± 0.025	0.252 ± 0.026	0.284 ± 0.023	0.126 ± 0.004	0.125 ± 0.004	0.097 ± 0.004	0.247 ± 0.006	0.149 ± 0.007	0.130 ± 0.022	0.151 ± 0.033	0.200 ± 0.021	5,942	5,521	244	177
Kanater	Wild	50	297.3-Infinite	0.216 ± 0.025	0.214 ± 0.025	0.215 ± 0.027	0.305 ± 0.025	0.221 ± 0.024	0.218 ± 0.024	0.241 ± 0.026	0.290 ± 0.022	0.153 ± 0.006	0.152 ± 0.006	0.130 ± 0.007	0.292 ± 0.007	0.172 ± 0.008	0.152 ± 0.009	0.153 ± 0.028	0.205 ± 0.020	7,627	7,125	316	186
Asyut	Wild	33	149.3-Infinite	0.268 ± 0.042	0.266 ± 0.042	0.270 ± 0.042	0.314 ± 0.044	0.259 ± 0.036	0.257 ± 0.036	0.292 ± 0.037	0.287 ± 0.034	0.164 ± 0.005	0.163 ± 0.005	0.133 ± 0.006	0.301 ± 0.008	0.182 ± 0.013	0.149 ± 0.012	0.147 ± 0.036	0.205 ± 0.026	6,553	6,106	260	187
Aswan	Wild	28	55.7-Infinite	0.247 ± 0.032	0.245 ± 0.042	0.251 ± 0.034	0.290 ± 0.033	0.265 ± 0.031	0.264 ± 0.031	0.288 ± 0.031	0.290 ± 0.029	0.137 ± 0.003	0.136 ± 0.003	0.107 ± 0.004	0.277 ± 0.006	0.169 ± 0.009	0.152 ± 0.012	0.147 ± 0.041	0.203 0.203	5,995	5,581	228	186
Domestic		**470**	**470**	**0.208 ± 0.007**	**0.203 ± 0.007**	**0.271 ± 0.006**	**0.281 ± 0.007**	**0.228 ± 0.007**	**0.222 ± 0.007**	**0.330 ± 0.006**	**0.289 ± 0.007**	**0.175 ± 0.001**	**0.171 ± 0.001**	**0.218 ± 0.002**	**0.263 ± 0.002**	**0.184 ± 0.003**	**0.151 ± 0.006**	**0.246 ± 0.007**	**0.204 ± 0.006**	**9,234**	**8,609**	**439**	**186**
Wild		**351**	**351**	**0.177 ± 0.009**	**0.176 ± 0.009**	**0.147 ± 0.009**	**0.286 ± 0.008**	**0.165 ± 0.009**	**0.191 ± 0.009**	**0.185 ± 0.010**	**0.285 ± 0.008**	**0.035 ± 0.001**	**0.140 ± 0.001**	**0.111 ± 0.002**	**0.272 ± 0.002**	**0.166 ± 0.003**	**0.127 ± 0.008**	**0.150 ± 0.011**	**0.202 ± 0.008**	**8,577**	**7,979**	**412**	**186**

*The estimated effective population size for each sampling location and/or timepoint calculated using linkage disequilibrium. Reported lower bound and upper bound numbers reflect a 95% confidence interval calculated using the jackknife method, a non-parametric method at a minimum allele frequency of 0.05. The average observed heterozygosity (H_*o*_), expected heterozygosity (H_*e*_), multilocus heterozygosity (MLH), minor allele frequency (MAF), and the number of polymorphic loci per sampling location and per STRUCTURE population designation (*K* = 2; domestic genetic cluster, wild genetic cluster). Bold text represents results for the two genetic clusters identified in structure.*

The domestic populations, considered as a whole genetic cluster and individually, had a higher MLH overall than wild populations across three marker subsets (All, Neutral, and Directional; Table 2). Manzala Lagoon had the lowest MLH in all three marker sets (MLH_All_ = 0.145, MLH_Neutral_ = 0.130, and MLH_Directional_ = 0.151; Table 1). However, when only balancing outlier markers were analyzed, genetic diversity indices for all populations and genetic clusters were similar to one another (Table 2).

The number of polymorphic loci per population ranged between 5,995 and 9,291 loci (61.0 – 94.5%), with domestic populations having 24.8% more polymorphic loci on average than the wild populations when all markers were considered (Table 2). A total of 565 private SNPs were identified within the domestic genetic cluster, while no private SNPs were identified within the wild genetic cluster.

As the number of polymorphic loci varied greatly between domestic and wild populations, the effect of missing data on genetic diversity indices was also examined (Supplementary Material 9). Markers with less than 50%, 25%, and 5% missingness in all samples were tested, as well as markers with a maximum of 5% missingness within a single population. As the percentage of missingness allowed per SNP decreased, the number of markers that passed this quality control measure also decreased. The number of polymorphic markers decreased from 61 to 95% when all markers were included to 44 – 67% when 50% missing data was allowed (Supplementary Material 9). The percentage of polymorphic markers was similar between 25% missing data (29–44%) and 5% missing data per population (27 – 48%; Supplementary Material 9).

In general, as the proportion of missing data allowed decreased, the number of polymorphic loci also decreased and estimates of observed and expected heterozygosity remained similar (±0.01) or decreased, with the exception of Rosetta at 25% missing data (Supplementary Material 9). The marker set with only a total of 5% missingness per population allowed had the lowest number of polymorphic markers (8.5 – 16.4%), H_o_, and H_e_ (Supplementary Material 9). In population groupings with a larger number of individuals sampled (121 – 470 samples), heterozygosity estimates were less affected and patterns remained more consistent than in groupings with fewer sampled individuals (28 - 50 samples; Supplementary Material 9). Rosetta (48 samples), Asyut (33 samples), and Aswan (20 samples) showed the greatest variability among marker subsets (Table 2 and Supplementary Material 9).

## Discussion

This study used genome-wide SNP markers to (1) investigate population genetic structure, (2) detect signatures of selection in three generations of the AS and eight wild populations of Nile tilapia (*O. niloticus*; Aswan, Manzala Lagoon, Kanater, Lake Idku, Damietta, Lake Burullus, Rosetta, and Asyut) throughout the Nile River, Egypt, and (3) audit genetic diversity in the AS and wild populations.

Clear population genetic structuring was observed indicating that the domesticated AS genetic cluster has become genetically distinct from the wild genetic cluster in Egypt. The genetic distinction between the AS and wild populations is likely due to the initial bottleneck created by a small founding population, genetic drift and the subsequent selection for faster growth rates, larger sizes, and domestication within this limited population. This clear separation between wild and domestic populations has also been observed in Atlantic Salmon, *Salmo salar* (Gutierrez et al., 2016) and gilthead sea bream, *Sparus aurata* (Cossu et al., 2019). The effects of the bottleneck created by the small founding population for AS can be observed in the smaller effective population size (max. 48.6) of the domesticated AS in comparison to the wild effective population size (max “infinite”). Similar results have been seen in other aquaculture species, like Atlantic Salmon, *Salmo salar* (Domestic Ne 33–125, Wild Ne = 50- >20,000; Bentsen and Thodesen, 2005), Pacific oyster, *Crassostrea gigas* (Domestic Ne = 47.6–58.5, Wild Ne = 527.9-infinite; Zhong et al., 2017), and gilthead sea bream, *Sparus aurata* (Domestic Ne = 21–111, Wild Ne = 133-infinity with the exception of one domestic population; Cossu et al., 2019).

The genetic difference between the AS and Aswan, one of the strain’s founding populations was one of the largest observed. This is surprising, but not entirely unexpected given the AS’s management history. A previous study found that the AS was created by two founding events with *O. niloticus* (Nayfa et al., 2020) in addition to the hybridization events with *O. aureus* (Grobler, 2017). Of the original founders, which included individuals from Aswan, only 53 of the original 201 founder genomes are present in Generations 9, 10, and 11 of the AS. From those 53 founder genomes, only 34 account for over 84% of the AS’ genetic composition (Nayfa et al., 2020). Thus, it is likely that the Aswan founder genomes have been bred out of the AS.

Despite evidence of gene flow among the eight wild populations, isolation-by-distance was detected with the two most southern populations (Asyut and Aswan) being more distinct from the Nile Delta populations to the north than the geographically intermediate Kanater population. In addition to the effects of physical distance to gene flow and population structure, environmental factors may have also influenced this distinction between Delta and upstream riverine populations. Individuals within Delta populations, particularly Lake Idku, Lake Burullus, and Manzala Lagoon, which have a direct connection to the sea, live in brackish to freshwater conditions whilst the individuals within the upstream populations live in freshwater conditions (Hassanien et al., 2004; Balah, 2012).

These results are similar to those observed in 2004 and 2005 in two separate studies using microsatellites and randomly amplified polymorphic DNA (RAPD) where evidence of population sub-structuring was identified (Hassanien et al., 2004; Hassanien and Gilbey, 2005). Structuring in these studies was not only identified between geographically distant Nile Delta populations and upstream Egyptian Nile populations, but also amongst lake and river base populations in the Delta (Hassanien et al., 2004; Hassanien and Gilbey, 2005). However, unlike those studies, the present study observed no significant population structuring among Nile Delta populations. This disparity may be attributed to the difference in molecular technologies utilized between studies and the dramatic rise in aquaculture in Egypt (Soliman and Yacout, 2016).

Differences in molecular technologies have likely contributed to the disparities in population structure. For instance, Hassanien and Gilbey (2005) inferred the presence of null alleles based on lower levels of observed vs. expected heterozygosity levels in their microsatellite dataset. Null alleles in microsatellite studies can result in the overestimation of *F*_*st*_ and genetic distance (Chapuis and Estoup, 2006). Whereas, the RAPDs used in Hassanien et al. (2004) are limited by the fact that the majority of RAPD markers are dominant, making it impossible to determine whether a DNA segment is amplified from a homozygous or heterozygous locus (Kumar and Gurusubramanian, 2011). This can result in uncertain estimates to genetic structure (Fritsch and Rieseberg, 1996). Additionally, the molecular criteria which determine what constitutes population structure are flexible and can vary based on the organism, study question, and genetic markers used (Waples and Gaggiotti, 2006; Putman and Carbone, 2014).

Genetic technologies are not the only factor to have changed over the years. Since 2005, Egypt has experienced a considerable increase in extensive, semi-intensive, and intensive farming systems for Nile tilapia (Soliman and Yacout, 2016). The vast majority of these farms are located in the Nile Delta region and concentrated in the Northern Lakes (Maruti, Idku, Brulus, and Manzala Lagoon; Soliman and Yacout, 2016). As a result, increased movement of fish among hatcheries and farms has occurred in the region in that time. In addition, the number of fish escaping from farms has likely increased due to a combination of local weather conditions, including flash flooding events (Moawad et al., 2016), and farm practices. With five of the eight sampled locations in the Nile Delta regions, and farming occurring at or near the remaining three sampling locations (Soliman and Yacout, 2016), the genetic diversity of the wild populations may have been affected by exchange with farmed stocks.

A comparison of wild and domestic genetic clusters identified 674 outlier markers, with a higher proportion of markers deviating from HWE in domestic populations than wild populations. This is indicative of a finite population size and selective forces, such as artificial selection for marketable traits and domestication (Waples, 2014). The large amount of outliers detected concurs with other genetic studies of domestic vs. wild aquatic populations, including brown trout *Salmo trutta* L., (431 SNP outliers; Linløkken et al., 2017) and Atlantic salmon, *Salmo salar* L. (337 and 270 SNP outliers; López et al., 2019). Both balancing and diversifying outliers identified between domestic and native populations were found in every chromosome. Unlike other studies which found specific regions of the genome under selection when comparing domestic and wild populations (Marrano et al., 2018; López et al., 2019), there was a lack of localized clustering of outliers.

A limited number of outliers (0 – 11) detected in pairwise comparisons of wild populations is consistent with the limited genetic differentiation observed among the wild populations. The fact that the number of outliers detected increased with geographic distance from the upstream (Asyut, Kanater, and Aswan) to Nile Delta populations (Lake Idku, Rosetta, Lake Burullus, Damietta, and Manzala Lagoon) also reflects the isolation-by-distance determined using the whole data set. These results suggest that despite known differences in salinity levels in delta and upstream populations, there appears to be little or no effect on selection. This is not entirely surprising as Nile tilapia are known for their tolerance to a wide range of environmental conditions (Balarin, 1982; Avella et al., 1993; Shelton and Popma, 2006; Rebouças et al., 2016). Alternatively gene flow may be high enough between geographic regions to combat the forces of natural selection (Lenormand, 2002). Consequently, few outliers amongst wild populations indicate that the AS would be expected to perform similarity in different locations once disseminated throughout Egypt.

Differences in genetic diversity resulted in the domesticated AS being clearly distinguishable from wild populations. In general, genetic diversity indices indicate that AS populations have higher levels of heterozygosity than wild populations. This held true regardless of the number of SNPs and levels of missing data allowed. These results differ from what is traditionally seen in domesticated and/or selectively bred populations vs. wild populations where wild populations exhibit either higher levels of genetic diversity (Makino et al., 2018; Zamani et al., 2018), or similar levels of heterozygosity (Gutierrez et al., 2016). This may be explained by (1) hybridization with another tilapia species, (2) the isolation-by-distance observed in this study among current wild populations and (3) the historical development of fishing and aquaculture in Egypt.

The AS had a higher number of polymorphic markers and private SNPs (5.7% of all SNPs) than wild populations. While this may be a result of domestication or founder effects, it is suspected that introgression has occurred with blue tilapia (*O. aureus*). Blue tilapia from a population maintained at the Abbassa Station, Egypt have been observed in earthen ponds in AS facilities (Benzie, 2019; pers. comm.). This population of blue tilapia has now been removed from the Abbassa Station. Unpublished research by the WorldFish Center and affiliated researchers found that the AS is comprised of 10% *O. aureus* (blue tilapia; Grobler, 2017). This interpretation is further supported by the large number of outliers detected, as hybridization has been interpreted to explain the detection of outliers in other species (Cullingham et al., 2014) and species-specific SNPs are often picked up when developing SNPs from samples that include multiple species or hybrids (Liu et al., 2011; Silva−Junior et al., 2015). Thus, the incorporation of *O. aureus* in the AS genome may account for the high number of private SNPs identified in the AS, as well as the higher number of polymorphic markers and heterozygosity observed in the AS genetic cluster over the wild genetic cluster as these markers may have been species-specific SNPs. While the AS showed the greatest number of polymorphic loci, the wild populations all exhibited different subsets of polymorphic loci per sampling location, indicating that hybridization with *O. aureus* may have also occurred in the wild. Given that tilapia species are well known for hybridizing in both aquaculture and wild environments, this is unsurprising (Lovshin, 1982; D’Amato et al., 2007; Deines et al., 2014; Meier et al., 2019).

Despite the low level of genetic distinction between wild and domestic populations of Nile tilapia detected in the present research, putative AS escapees were easily identified, with suggested evidence of first and later generation escapees in Rosetta, Kanater, and Damietta detected. Escapees in other locales, particularly from selectively bred individuals, have been shown to lower the fitness of wild populations (Yang et al., 2019) as demonstrated in Atlantic salmon, *Salmo salar* (McGinnity et al., 2003; Glover et al., 2013); European sea bass, *Dicentrarchus labrax* (Toledo-Guedes et al., 2014); and Turbot, *Scophthalmus maximus* (Prado et al., 2018). It is not clear to what extent this may be a concern for tilapia, because while there was evidence in the present study of AS genetic material in wild Egyptian populations, to date, there is no information on fitness differentials between the domesticated AS and wild tilapia populations.

High levels of genetic diversity were still observed within the AS, suggesting that the potential detrimental effects on diversity of any AS escapees that do survive in wild populations may be minimal. This is particularly true as the AS was founded from both Nile Delta and upstream populations of Nile tilapia in Egypt. Thus, the genetic diversity observed in the AS is a subset of what is already available in wild populations. This in addition to the relatively low number of escapees detected when considering all wild populations, suggests that escapees may either be a rare occurrence or may have low survival within wild populations. This has been demonstrated previously in domestic rainbow trout (*Oncorhynchus mykiss*) who experience lower survival rates in the wild due to their increased size and bolder foraging habits exposing them to higher predation (Biro et al., 2004). Regardless, continued monitoring of escapees from the AS and other domestic lines is important as many wild Nile tilapia populations are at risk of an altered population structure and genetic diversity due to anthropogenic changes such as habitat disturbance, overfishing, and indiscriminate fish transfers of tilapia species throughout Africa (Eknath and Hulata, 2009).

## Conclusion

The present study has highlighted the valuable information for improved management of aquaculture species by investigating population genetic structure, genetic diversity, and signatures of selection between domestic and wild populations. In the case of Nile tilapia in Egypt, domestic and wild populations were found easily distinguishable from one another using SNP markers, even when compared to founding populations. In turn, this distinct clustering allowed for easy detection of putative escapees. Although the wild genetic cluster was not panmictic, with wild populations displaying evidence of isolation-by-distance, levels of genetic differentiation were relatively low and no evidence of significant signatures of selection among wild populations were observed. After 11 years of selective breeding, the AS displayed high levels of genetic diversity. These data suggest that the AS could be disseminated throughout Egypt with negligible differences in performance expected and minimal disruption to wild populations. The genetic diversity comparisons also helped better understand how the effects of selection, founder effect, inbreeding, and genetic drift have affected this domestic line. The introgression with *O. aureus* may explain the large number of outliers detected between wild and captive genetic clusters. While both balancing and diversifying outliers were traced back to all 22 *O. niloticus* chromosomes, additional research is required to determine the nature of these signatures and their direct relevance to biological or evolutionary processes within domestic and wild populations.

## Data Availability Statement

The datasets presented in this study can be found in online repositories. The names of the repository/repositories and accession number(s) can be found below: https://doi.org/10.7910/DVN/IFSGQF, https://dataverse.harvard.edu/dataset.xhtml?persistentId=doi:10.7910/DVN/IFSGQF.

## Ethics Statement

Ethical review and approval was not required for the animal study because finclips for genetic sampling were acquired from a commercial farm during harvesting and from fishing boat catches. Written informed consent was obtained from the owners for the participation of their animals in this study.

## Author Contributions

MN conceptualized the project, acquired partial project funding, led the acquisition and formal analysis of the data as well as led the literature research and writing of the manuscript. DBJ supervised the data analysis and contributed to the review and editing of the manuscript. JB helped to conceptualize the project, acquired the majority of project funding, supervised the data acquisition, and contributed to the review and editing of the manuscript. KZ helped to conceptualize the project, supervised the execution of the project and analysis of data as well as contributed to the review and editing of the manuscript. DRJ helped to conceptualize the project, supervised the research project, acquired partial project funding, and contributed to editing the manuscript. All authors contributed to the article and approved the submitted version.

## Conflict of Interest

The authors declare that the research was conducted in the absence of any commercial or financial relationships that could be construed as a potential conflict of interest.

## Footnotes

1 https://www.diversityarrays.com

2 http://www.kddart.org/kdcompute.html

3 https://github.com/esteinig/dartqc
