# Supplementary material for: Comparing Genomic Signatures of Selection Between the Abbassa Strain and Eight Wild Populations of Nile Tilapia (Oreochromis niloticus) in Egypt
Source: Front Genet. 2020 Oct 15;11:567969. doi: 10.3389/fgene.2020.567969 (PMC7593532; doi:10.3389/fgene.2020.567969)
Supplement: Supplementary file 1 [file Data_Sheet_1.zip › SupplementaryMaterial/SupplementaryMaterial_2.pdf]

Comparing genomic signatures of selection between the Abbassa Strain and eight wild populations of Nile tilapia (*Oreochromis niloticus*) in Egypt

Maria G. Nayfa[1,2*], David B. Jones[1,2], John A.H. Benzie[3,5], Dean R. Jerry[1,2,4], and Kyall R. Zenger[1,2]

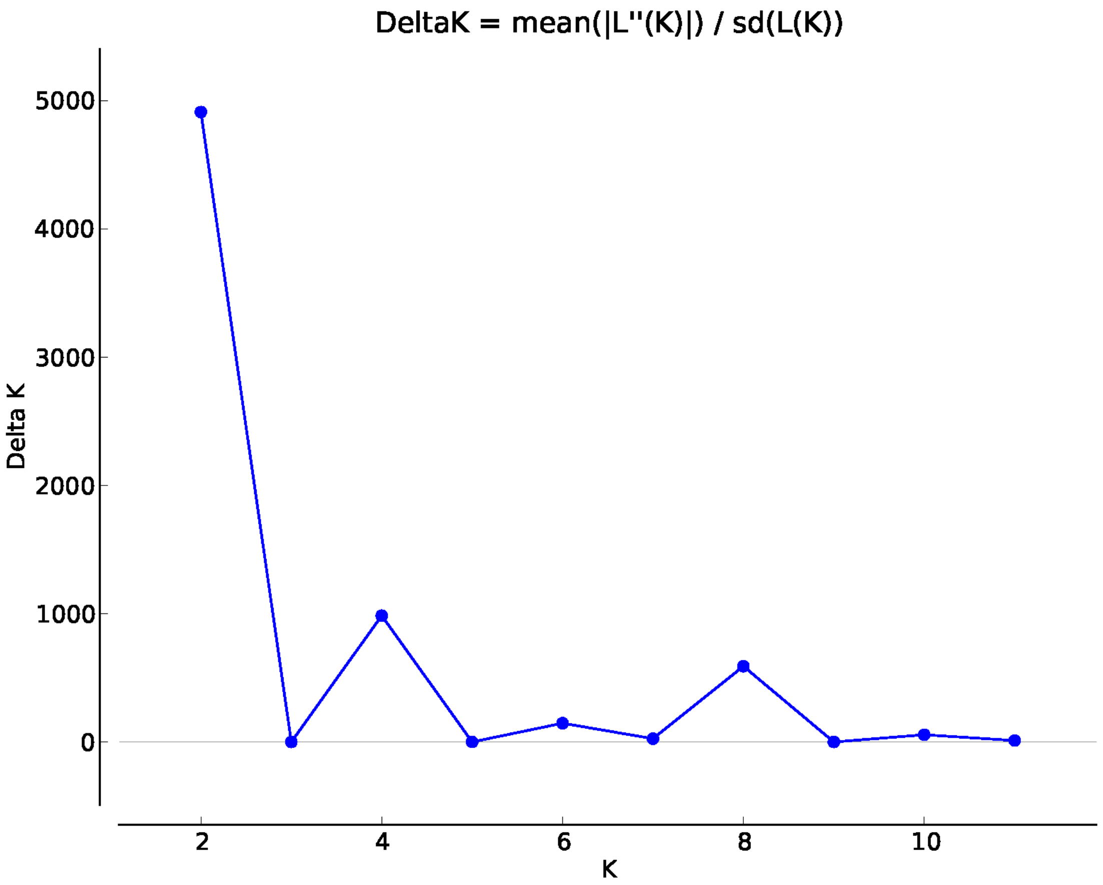

Supplementary Material 2. Evanno ΔK values calculated for all 11 potential populations sampled (3 generations of ASL of Nile tilapia; 8 sampling locations of natural Nile tilapia, *O. niloticus*). Results are based on 3 iterations of K1-12.
