# Supplementary material for: Comparing Genomic Signatures of Selection Between the Abbassa Strain and Eight Wild Populations of Nile Tilapia (Oreochromis niloticus) in Egypt
Source: Front Genet. 2020 Oct 15;11:567969. doi: 10.3389/fgene.2020.567969 (PMC7593532; doi:10.3389/fgene.2020.567969)
Supplement: Supplementary file 1 [file Data_Sheet_1.zip › SupplementaryMaterial/SupplementaryMaterial_3.pdf]

Comparing genomic signatures of selection between the Abbassa Strain and eight wild populations of Nile tilapia (*Oreochromis niloticus*) in Egypt

Maria G. Nayfa[1,2*], David B. Jones[1,2], John A.H. Benzie[3,5], Dean R. Jerry[1,2,4], and Kyall R. Zenger[1,2]

Supplementary Material 3. Pairwise comparison of genetic distance (F_{st}) values for all three generations of the ASL and all eight natural populations of *O. niloticus* . Significant F_{st} values with a p-value < 0.05 are indicated by an asterix (*).

	Gen 9	***Gen 10***	***Gen 11***	***Aswan***	***Manzala Lagoon***	***Kanater***	***Lake Idku***	***Damietta***	***Lake Burullus***	***Rosetta***	***Asyut***
Gen 9	0										
Gen 10	-0.008	0									
Gen 11	-0.002	-0.004	0								
Aswan	0.045*	0.048*	0.050*	0							
Manzala Lagoon	-0.003	0.006*	0.017*	0.006*	0						
Kanater	0.035*	0.034*	0.042*	0.014*	-0.015	0					
Lake Idku	0.007*	0.011*	0.027*	0.011*	0.003*	-0.006	0				
Damietta	0.024*	0.025*	0.038*	0.020*	-0.009	0.002*	0.003*	0			
Lake Burullus	0.034*	0.035*	0.046*	0.021*	-0.013	0.006*	-0.005	0.001*	0		
Rosetta	0.024*	0.023*	0.032*	0.013*	-0.023	-0.002	-0.012	-0.009	0.000	0	
Asyut	0.055*	0.052*	0.058*	0.003*	-0.012	0.006*	-0.005	0.012*	0.014*	0.000	0
