# Supplementary material for: Comparing Genomic Signatures of Selection Between the Abbassa Strain and Eight Wild Populations of Nile Tilapia (Oreochromis niloticus) in Egypt
Source: Front Genet. 2020 Oct 15;11:567969. doi: 10.3389/fgene.2020.567969 (PMC7593532; doi:10.3389/fgene.2020.567969)
Supplement: Supplementary file 1 [file Data_Sheet_1.zip › SupplementaryMaterial/SupplementaryMaterial_4.pdf]

Comparing genomic signatures of selection between the Abbassa Strain and eight wild populations of Nile tilapia (*Oreochromis niloticus*) in Egypt

Maria G. Nayfa[1,2*], **David B. Jones**[1,2], **John A.H. Benzie**[3,5], **Dean R. Jerry**[1,2,4], **and Kyall R. Zenger**[1,2]

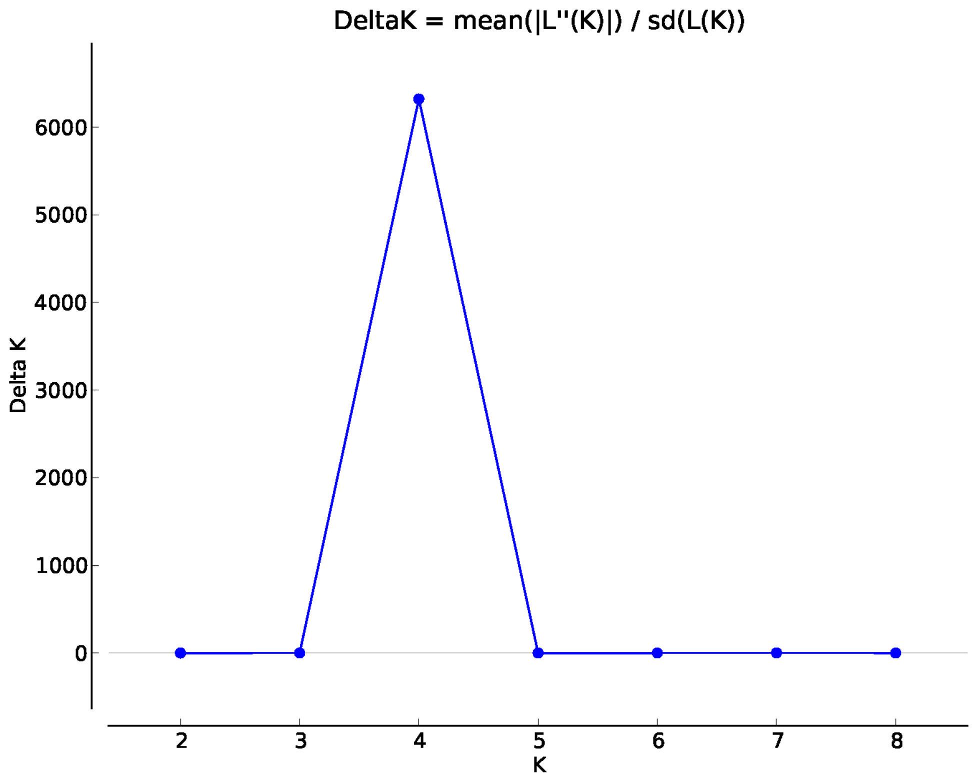

Supplementary Material 4. Evanno ΔK values calculated for all 8 sampling locations of natural Nile tilapia, *O. niloticus*. Results are based on 3 iterations of K1-9.
