# Supplementary material for: Comparing Genomic Signatures of Selection Between the Abbassa Strain and Eight Wild Populations of Nile Tilapia (Oreochromis niloticus) in Egypt
Source: Front Genet. 2020 Oct 15;11:567969. doi: 10.3389/fgene.2020.567969 (PMC7593532; doi:10.3389/fgene.2020.567969)
Supplement: Supplementary file 1 [file Data_Sheet_1.zip › SupplementaryMaterial/SupplementaryMaterial_5.pdf]

Comparing genomic signatures of selection between the Abbassa Strain and eight wild populations of Nile tilapia (*Oreochromis niloticus*) in Egypt

Maria G. Nayfa[1,2*], David B. Jones[1,2], John A.H. Benzie[3,5], Dean R. Jerry[1,2,4], and Kyall R. Zenger[1,2]

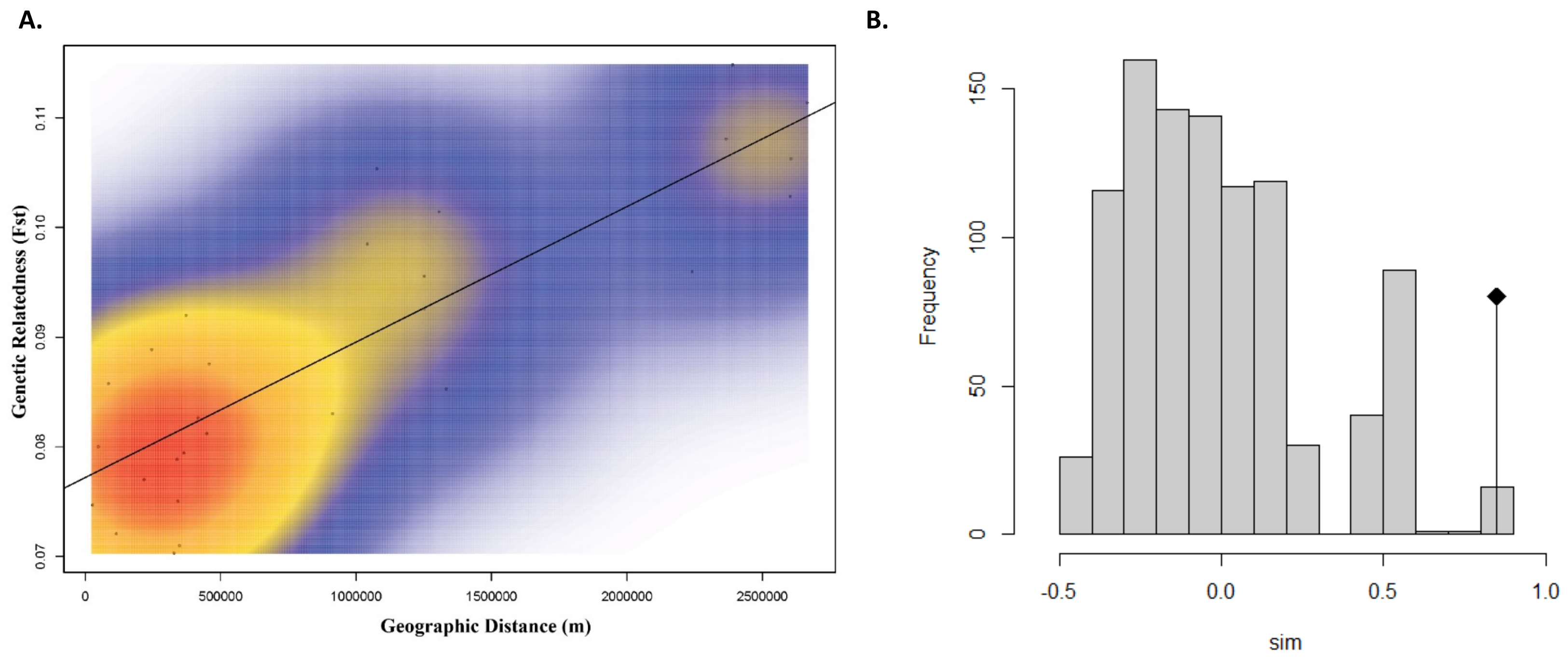

Supplementary Material 5. Isolation-by-Distance. A) A heatmap comparing genetic relatedness (F_{st}) between two populations to their geographical distance (km) from one another. The heat map runs on a scale of red (higher relatedness). B) Histogram of sim testing representing the results from a Mantel test between matrices of genetic and geographic distances to test for isolation-by-distance (simulated p-value= 0.003). The original value of correlation between the two matrices is represented by the line and diamond. The histogram represents the simulated values when spatial structure is absent.
