# Supplementary material for: Comparing Genomic Signatures of Selection Between the Abbassa Strain and Eight Wild Populations of Nile Tilapia (Oreochromis niloticus) in Egypt
Source: Front Genet. 2020 Oct 15;11:567969. doi: 10.3389/fgene.2020.567969 (PMC7593532; doi:10.3389/fgene.2020.567969)
Supplement: Supplementary file 1 [file Data_Sheet_1.zip › SupplementaryMaterial/SupplementaryMaterial_6.pdf]

Comparing genomic signatures of selection between the Abbassa Strain and eight wild populations of Nile tilapia (*Oreochromis niloticus*) in Egypt

Maria G. Nayfa[1,2*], David B. Jones[1,2], John A.H. Benzie[3,5], Dean R. Jerry[1,2,4], and Kyall R. Zenger[1,2]

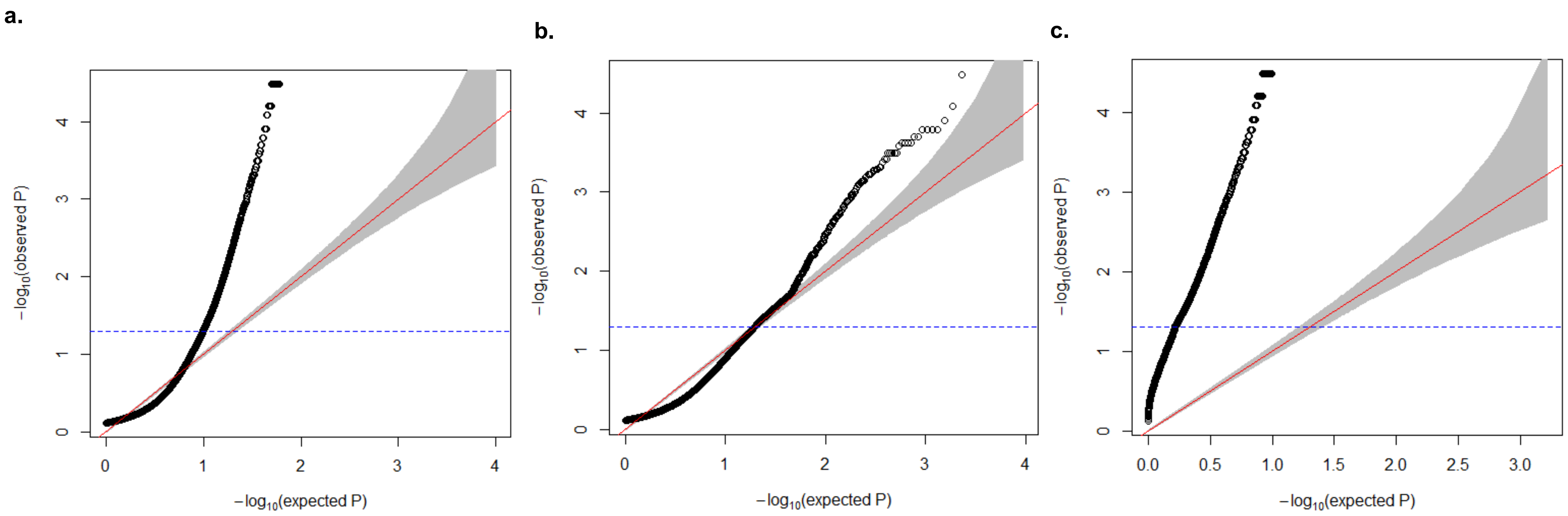

Supplementary Material 6. Quantile-Quantile (QQ) Plots of (a) all loci (b) neutral loci (i.e. where both balancing and directional outliers jointly identified by BayeScan v. 2.1 and Arlequin v. 3.5.2.2 were removed), and (c) neutral$_{\text{all outliers}}$ loci (i.e. where any balancing and directional outliers identified in either BayeScan v. 2.1 or Arlequin v. 3.5.2.2 were removed). For each of the figures above the dotted blue line indicates the significance threshold of outliers identified at $p \leq 0.05$, the red line represent normally distributed data where the observed and expected p-value distributions are equivalent, and the surrounding grey area represents a 95% confidence interval.
