# Supplementary material for: Comparing Genomic Signatures of Selection Between the Abbassa Strain and Eight Wild Populations of Nile Tilapia (Oreochromis niloticus) in Egypt
Source: Front Genet. 2020 Oct 15;11:567969. doi: 10.3389/fgene.2020.567969 (PMC7593532; doi:10.3389/fgene.2020.567969)
Supplement: Supplementary file 1 [file Data_Sheet_1.zip › SupplementaryMaterial/SupplementaryMaterial_8.pdf]

Comparing genomic signatures of selection between the Abbassa Strain and eight wild populations of Nile tilapia (*Oreochromis niloticus*) in Egypt

Maria G. Nayfa[1,2*], David B. Jones[1,2], John A.H. Benzie[3,5], Dean R. Jerry[1,2,4], and Kyall R. Zenger[1,2]

Supplementary Material 8. Genetic diversity indicies calculated using all SNPs. Hardy-Weinberg Equilibrium (HWE) was calculated as the proporiton of markers that were significantly (p-value < 0.05) out of HWE, Monomorphic SNPs were calculated as the porportion of markers that were monomorphic, and the inbreeding coeffiient (F_{is}) was calculated per sampling location, timepoint, and/or population. Significant F_{is} values are denoted by *.

	Category	*n*	*HWE*	*Monomorphic SNPs*	F_{is}
Gen 9	Domestic	121	0.114	0.019	-0.035
Gen 10	Domestic	204	0.138	0.020	-0.078
Gen 11	Domestic	145	0.146	0.031	-0.054
Lake Idku	Natural	49	0.030	0.252	-0.067
Rosetta	Natural	48	0.041	0.144	0.038
Lake Burullus	Natural	50	0.032	0.263	-0.055
Damietta	Natural	50	0.041	0.213	-0.027
Manzala Lagoon	Natural	43	0.023	0.288	-0.090
Kanater	Natural	50	0.025	0.181	-0.112
Asyut	Natural	33	0.010	0.326	-0.142
Aswan	Natural	28	0.015	0.355	-0.065
Domestic		470	0.210	0.008	-0.060
Natural		351	0.194	0.057	-0.061
