# Supplementary material for: Comparing Genomic Signatures of Selection Between the Abbassa Strain and Eight Wild Populations of Nile Tilapia (Oreochromis niloticus) in Egypt
Source: Front Genet. 2020 Oct 15;11:567969. doi: 10.3389/fgene.2020.567969 (PMC7593532; doi:10.3389/fgene.2020.567969)
Supplement: Supplementary file 1 [file Data_Sheet_1.zip › SupplementaryMaterial/SupplementaryMaterial_9.pdf]

Comparing genomic signatures of selection between the Abbassa Strain and eight wild populations of Nile tilapia (*Oreochromis niloticus*) in Egypt

Maria G. Nayfa[1,2*], David B. Jones[1,2], John A.H. Benzie[3,5], Dean R. Jerry[1,2,4], and Kyall R. Zenger[1,2]

Supplementary Material 9. A subset of genetic diversity indices-including, average observed heterozygosity (H_o), expected heterozygosity (H_e), and the number of polymorphic loci- calculated using different allowances of missingness in data per sampling location and per STRUCTURE population designation (K = 2; domestic population, natural population). All loci included, all loci potentially included in analysis with loci varying per population with only 5% missingness allowed within each population (All Loci; 5% Missing Allowed Per Population), markers present in at least 50% of samples allowed (50% missingness), markers present in at least 75% of samples allowed (25% missingness), and markers present in at least 95% of samples allowed (5% missingness).

	H_O ± SE					H_E ± SE					Polymorphic Loci				
	All Loci	*All Loci: 5% Missing Allowed Per Population*	*50%*	*25%*	*5%*	*All Loci*	*All Loci: 5% Missing Allowed Per Population*	*50%*	*25%*	*5%*	*All Loci*	*All Loci: 5% Missing Allowed Per Population*	*50%*	*25%*	*5%*
**Gen9**	0.211±0.013	0.221±0.014	0.219±0.013	0.222±0.014	0.185±0.012	0.232±0.014	0.185±0.011	0.233±0.014	0.230±0.014	0.185±0.011	9,291	4519	6527	4297	1594
**Gen10**	0.212±0.010	0.220±0.011	0.218±0.010	0.219±0.010	0.182±0.009	0.231±0.011	0.183±0.009	0.233±0.011	0.229±0.011	0.183±0.009	8,671	3734	6497	4290	1591
**Gen11**	0.211±0.013	0.219±0.013	0.218±0.013	0.218±0.013	0.183±0.011	0.229±0.013	0.181±0.011	0.230±0.013	0.226±0.013	0.181±0.011	8,934	4298	6449	4257	1584
**Lake Idku**	0.214±0.024	0.211±0.024	0.214±0.024	0.206±0.024	0.151±0.019	0.232±0.024	0.153±0.019	0.228±0.024	0.216±0.024	0.153±0.019	6,404	3484	5143	3496	1107
**Rosetta**	0.181±0.029	0.178±0.024	0.181±0.024	0.271±0.028	0.115±0.020	0.212±0.029	0.133±0.018	0.209±0.024	0.253±0.028	0.133±0.018	7,626	4687	5800	2902	1368
**Lake Burullus**	0.221±0.024	0.214±0.024	0.221±0.024	0.216±0.026	0.153±0.020	0.236±0.024	0.154±0.019	0.232±0.024	0.211±0.024	0.154±0.019	6,754	3844	4960	3634	1045
**Damietta**	0.202±0.023	0.204±0.023	0.203±0.023	0.225±0.024	0.143±0.019	0.225±0.024	0.144±0.019	0.220±0.024	0.232±0.023	0.144±0.019	7,041	3752	5306	3249	1170
**Manzala Lagoon**	0.222±0.025	0.223±0.026	0.223±0.026	0.182±0.026	0.162±0.022	0.240±0.025	0.161±0.0210	0.237±0.025	0.205±0.025	0.161±0.021	5,942	2896	4922	3838	1037
**Kanater**	0.216±0.025	0.191±0.024	0.218±0.026	0.222±0.024	0.139±0.021	0.221±0.024	0.134±0.019	0.218±0.024	0.227±0.023	0.134±0.019	7,627	3591	5474	3259	1254
**Asyut**	0.268±0.042	0.218±0.032	0.272±0.034	0.254±0.031	0.185±0.028	0.259±0.036	0.175±0.0250	0.258±0.029	0.258±0.028	0.175±0.025	6,553	3004	4442	2858	843
**Aswan**	0.247±0.032	0.246±0.033	0.252±0.033	0.214±0.031	0.184±0.028	0.265±0.031	0.184±0.026	0.263±0.031	0.223±0.031	0.184±0.026	5,995	2693	4321	3380	839
**Domestic**	0.208±0.007	0.219±0.007	0.216±0.007	0.218±0.007	0.181±0.006	0.228±0.007	0.181±0.006	0.230±0.007	0.226±0.007	0.181±0.006	9,234	3822	6569	4326	1610
**Natural**	0.177±0.009	0.170±0.009	0.178±0.009	0.179±0.009	0.110±0.007	0.165±0.009	0.113±0.007	0.190±0.009	0.186±0.009	0.113±0.007	8,577	4008	6238	4098	1486
